# Overlapping and distinct fatty acid dysregulation in infertility and recurrent spontaneous abortion

**DOI:** 10.3389/fendo.2026.1866902

**Published:** 2026-06-12

**Authors:** Siqi Wei, Yuanxing Li, Pengdan Luo, Xinyu Yang, Peng-Sheng Zheng

**Affiliations:** 1 Department of Reproductive Medicine, The First Affiliated Hospital of Xi’an Jiaotong University, Xi’an, Shaanxi, China; 2 Assisted Reproduction Center, Northwest Women’s and Children’s Hospital, Xi’an, Shaanxi, China; 3 Department of Obstetrics and Gynecology, The First Affiliated Hospital of Xi’an Jiaotong University, Xi’an, Shaanxi, China

**Keywords:** fatty acid, infertility, recurrent spontaneous abortion, antiphospholipid antibodies, PUFAs

## Abstract

**Introduction:**

Infertility and recurrent spontaneous abortion (RSA) are common reproductive disorders with rising incidence. Both fatty acid (FA) metabolism and antiphospholipid antibodies (aPL) are known to be associated with reproductive outcomes. However, their interaction and distinct roles in infertility versus RSA remain poorly understood.

**Methods:**

This retrospective study included 388 women (64 controls, 135 infertility cases, 189 RSA cases). FA profiles were measured from dried blood spots by gas chromatography. Candidate FAs were identified by FDR−corrected univariate analyses and refined by random forest. Core FAs were defined as the intersection of significant FAs and random forest–important variables. Associations between core FAs and aPL positivity were assessed by logistic regression. Mediation analysis used structural equation modeling.

**Results:**

An elevated omega−6/omega−3 ratio and lower DPAn3 were seen in both infertility and RSA. Infertility also showed a broader decline in omega−3−related FAs (omega−3, EPA, DHA; all *P* < 0.05), while RSA featured higher behenic acid (*P* = 0.023). The omega−6/omega−3 ratio was positively associated with aPL positivity in both groups (infertility: adjusted OR = 3.51, 95% CI 2.24 – 5.91, *P* < 0.001; RSA: OR = 4.87, 95% CI 3.15 – 8.05, *P* < 0.001); DPAn3 was inversely associated with aPL positivity in both groups (infertility: *P* < 0.05; RSA: adjusted OR = 0.33, 95% CI 0.18 – 0.53, *P* < 0.001). In mediation analysis, aPL positivity partially mediated the relationship between the omega−6/omega−3 ratio (indirect effect 0.110, 30.7% mediated, *P* = 0.045) and DPAn3 (indirect effect 0.080, 40.1% mediated, *P* = 0.022) with RSA, but not with infertility.

**Discussion:**

Infertility and RSA exhibit overlapping yet distinct FA profiles. aPL positivity partially mediates the associations of the omega−6/omega−3 ratio and DPAn3 with RSA, but not with infertility. These findings demonstrate metabolic and immunological heterogeneity associated with different adverse reproductive outcomes.

## 1 Introduction

Infertility and recurrent spontaneous abortion (RSA) are common reproductive disorders among women of childbearing age, and their incidence has been increasing annually in recent years (1). According to a 2023 World Health Organization report, approximately 17.5% of the global adult population—roughly one in six individuals—is affected by infertility (2). The incidence of RSA ranges from 1% to 5%, and approximately 50% of cases remain unexplained despite thorough investigation (3) (4).

The role of fatty acid (FA) metabolism in reproductive health has received increasing attention in recent years. FAs are not only essential structural components of cell membrane phospholipids but also, through their derivatives, participate in the regulation of inflammation, oxidative stress, and immune cell function (5). The role of omega−3 and omega−6 polyunsaturated FAs (PUFAs) in female reproductive health has been extensively investigated.

Multiple observational studies and clinical trials indicate that higher omega−3 intake or circulating levels are associated with improved fertility. A cross−sectional study based on the NHANES database reported a positive association between the omega−6/omega−3 ratio and infertility risk among women aged 20–34 years (6). In women with polycystic ovary syndrome (PCOS)−related infertility, omega−3 supplementation improved the clinical pregnancy rate in overweight or obese participants (7), and those who achieved live birth had significantly higher serum DPA (includes two isomers: DPAn3 and DPAn6) and EPA levels than those who experienced pregnancy loss (8). Furthermore, a prospective cohort study found that self−reported omega−3 supplement use was associated with an increased probability of natural conception (9), and a systematic review suggested that dietary omega−3 intake may improve ART success rates by enhancing oocyte and embryo quality (10). In an animal model, fat−1 transgenic mice, which maintain a low tissue omega−6/omega−3 ratio, exhibited increased primordial follicle counts and improved reproductive outcomes, with serum DHA, DPA, and EPA levels positively correlated with follicle numbers (11).

With regard to RSA, existing evidence has largely focused on the relationship between omega−3 intake and pregnancy loss risk. An assisted reproductive technology (ART) cohort study found that higher EPA+DHA intake in women was associated with a lower risk of pregnancy loss (12). A first−trimester dietary analysis also reported that women with healthy pregnancies had higher omega−3 intake than those who experienced early pregnancy loss (13). In women with prior pregnancy loss, omega−3 supplementation significantly reduced triglyceride levels (14), suggesting a potential metabolic benefit. Nevertheless, studies examining circulating FA profiles in women with RSA remain limited, and the specific roles of DPA and the omega−6/omega−3 ratio in RSA have yet to be clarified.

Antiphospholipid antibodies (aPL) are a group of autoantibodies directed against phospholipids and phospholipid−binding proteins, and they represent the core laboratory markers for the diagnosis of antiphospholipid syndrome (APS) (15). aPL contribute to adverse pregnancy outcomes and thrombotic events primarily through endothelial activation, promotion of platelet aggregation, and disruption of trophoblast function (16) (17). Epidemiologically, aPL positivity is not restricted to APS; detectable aPL are found in approximately 1%–5% of the general healthy population and in up to 6% of pregnant women without diagnosed APS, with substantially higher positivity rates observed among women with RSA or infertility (16). At the molecular level, the major antigenic targets of aPL—including cardiolipin and β2−glycoprotein I—are intimately associated with phospholipids, and the core structure of phospholipids is composed of FAs. Consequently, FA composition can influence phospholipid conformation, cell membrane fluidity, and antigen presentation efficiency (17). Alterations in FA metabolism and circulating FA profiles may therefore participate in the generation and pathogenicity of aPL by modifying phospholipid architecture and modulating inflammatory and immune responses, positioning FA dysregulation as a potential metabolic contributor to autoimmune susceptibility (17).

Dry blood spot (DBS) sampling from fingertip blood—minimally invasive, requiring only microliter volumes, and stable at ambient temperature—has facilitated large-scale FA profiling in nutritional epidemiology (18) (19), yet its application in reproductive research remains scarce.

In this retrospective study, we compared FA profiles measured by DBS among women with infertility, women with RSA, and normal controls. Using a combination of multivariate screening, random forest ranking, and mediation analysis, we aimed to (i) identify FA signatures associated with each reproductive outcome, and (ii) evaluate whether aPL positivity mediates the associations between FAs and these outcomes.

## 2 Methods

### 2.1 Study design and participants

#### 2.1.1 Study setting and ethics approval

This retrospective clinical study was conducted at the First Affiliated Hospital of Xi’an Jiaotong University. Women undergoing fertility evaluation between October 2023 and March 2024 were consecutively recruited. The study protocol was approved by the Ethics Committee of the First Affiliated Hospital of Xi’an Jiaotong University (Approval No. XJTU1AF2022LSK-078) and adhered to the Declaration of Helsinki. Written informed consent was obtained from all participants prior to sample collection.

#### 2.1.2 Inclusion and exclusion criteria

Women aged 22–40 years were eligible. The following were excluded from all three groups: diagnosed autoimmune disease; diabetes mellitus; thyroid dysfunction requiring pharmacotherapy; use of hormonal medications, anticoagulants, or immunosuppressants within three months before enrollment; known parental karyotypic abnormalities; current smoking or alcohol consumption; and regular intake of omega−3 or other FA supplements within the preceding three months. All participants were instructed to maintain their usual dietary habits before blood collection.

Infertility group: The infertility group is defined by all of the following: inability to conceive after at least 2 years of regular unprotected intercourse; normal follicular development and ovulation confirmed by transvaginal ultrasound; no evidence of diminished ovarian reserve; bilateral tubal patency documented by hysterosalpingography or laparoscopy; and normal uterine cavity on three-dimensional ultrasound (absence of endometrial polyps, intrauterine adhesions, submucosal leiomyomas, and congenital anomalies). No male factor infertility was present (oligospermia, asthenospermia, azoospermia, and teratozoospermia were excluded).

RSA group: The RSA group is defined by all of the following: a history of two or more consecutive clinical pregnancy losses; normal uterine cavity morphology on hysteroscopy or three-dimensional ultrasound; if cytogenetic analysis of products of conception had been performed, losses attributable to embryonic aneuploidy were excluded; however, such testing was not systematically available for all cases.

NC: The NC group was defined as follows: women under 30 years of age were required to have no history of infertility or miscarriage; women over 30 years of age were required to have at least one previous term live birth and no history of infertility or miscarriage. All controls were neither pregnant nor lactating at the time of enrollment.

### 2.2 FAs profiling by DBS

Fasting capillary whole blood samples were collected in the morning following an overnight fast of at least 8 hours. Blood was applied directly onto specialized filter paper to generate DBS. Each blood spot was filled completely to ensure uniformity, and cards were air−dried at room temperature in a dark, low−humidity environment. Dried cards were individually sealed in airtight bags with desiccant and shipped at ambient temperature to a CNAS−accredited commercial laboratory (OmegaBandz. Inc., Shanghai, China) for analysis by gas chromatography (Agilent 7820; DB-23, 60-m column).

The FA panel included omega−3 PUFAs [α−linolenic acid (ALA, 18:3n−3), eicosapentaenoic acid (EPA, 20:5n−3), docosapentaenoic acid n−3 (DPAn3, 22:5n−3), docosahexaenoic acid (DHA, 22:6n−3)]; omega−6 PUFAs [linoleic acid (LA, 18:2n−6), γ−linolenic acid (GLA, 18:3n−6), dihomo−γ−linolenic acid (DGLA, 20:3n−6), arachidonic acid (AA, 20:4n−6), adrenic acid (22:4n−6), docosapentaenoic acid n−6 (DPAn6, 22:5n−6)]; cis-monounsaturated FAs (MUFAs) [tetradecenoic acid (14:1), pentadecenoic acid (15:1), palmitoleic acid (16:1), heptadecenoic acid (17:1), oleic acid (18:1n−9), eicosenoic acid (20:1n−9), erucic acid (22:1), nervonic acid (NA, 24:1n−9)]; and saturated FAs (SFAs) [myristic acid (14:0), pentadecanoic acid (15:0), palmitic acid (16:0), margaric acid (17:0), stearic acid (18:0), arachidic acid (20:0), behenic acid (22:0), lignoceric acid (24:0)].

Three derived indices were calculated as follows:

EPA/AA ratio = EPA (%)/AA (%).

Omega−6/Omega−3 ratio = (LA + GLA + DGLA + AA + adrenic acid + DPAn6)/(ALA + EPA + DPAn3 + DHA).

SFA/UFA ratio = (total SFAs)/(total UFAs, including both PUFAs and MUFAs).

### 2.3 Antiphospholipid antibody assessment

Fresh serum samples were used for aPL testing. IgA, IgM, and IgG antibodies against β2−glycoprotein I (anti−β2GPI) and cardiolipin (aCL) were measured using commercial ELISA kits (Euroimmun, Lübeck, Germany). IgA, IgM, and IgG antibodies against phosphatidylserine/prothrombin complex (anti−PS/PT) and IgM antibodies against vimentin/cardiolipin complex (V−ACA) were measured using a chemiluminescence immunoassay (CLIA) analyzer (BIO−FLASH, Werfen, Barcelona, Spain). For this analysis, aPL positivity was defined as at least one positive result among the tested antibodies according to the manufacturer’s reference ranges. Of note, aPL positivity herein reflects a single-test result and is not indicative of a definitive APS diagnosis.

### 2.4 Statistical analysis

All statistical analyses were performed in R version 4.4.1. Continuous variables are presented as mean ± standard deviation or median (interquartile range) depending on normality, and categorical variables as counts (percentages). Group comparisons of baseline characteristics were conducted using one−way ANOVA, Kruskal–Wallis test, or Fisher’s exact test as appropriate.

#### 2.4.1 Overall and univariate analyses of FAs profiles

To evaluate overall differences in the FA profile among the three groups, multivariate analysis of covariance (MANCOVA) was performed with Pillai’s trace as the test statistic, adjusting for age and BMI. Other potential confounders—such as smoking, alcohol consumption, and use of omega−3 or other FAs supplements—were explicitly excluded by the study’s exclusion criteria and therefore did not require adjustment.

Subsequently, individual FAs were assessed using analysis of covariance (ANCOVA) for normally distributed variables, or the Kruskal–Wallis test on age- and BMI-adjusted residuals for non-normally distributed variables. The resulting P values were corrected for multiple comparisons using the Benjamini–Hochberg false discovery rate (FDR) method. A screening threshold of q < 0.10 was applied to define the initial candidate FA set, hereafter referred to as Set_Sig.

#### 2.4.2 Random forest modeling and definition of core FAs

Two binary random forest models (RF1: infertility vs. NC; RF2: RSA vs. NC) were constructed using all FAs that passed univariate screening as candidate predictors. Continuous variables were centered and scaled, and categorical variables were converted to factors. Each forest was trained with 500 trees (mtry set to the default square root of the number of predictors; random seed 123). No explicit class−balancing was applied because the primary goal was variable importance ranking, and random forest is relatively robust to moderate class imbalance for this purpose. Variable importance was assessed by the mean decrease in Gini impurity. Variables were ranked in descending order of importance, and the point at which a marked drop (i.e., an elbow point, which was identified by visually inspecting the importance ranking curves as the positions where the marginal gain in importance dropped substantially) in the difference between adjacent importance scores occurred was identified.

As a sensitivity analysis to address class imbalance, we repeated the random forest procedures using synthetic minority over-sampling technique (SMOTE) (K = 5, dup_size = 0) to balance the training data. To further assess the robustness of variable importance, we also performed two additional sensitivity analyses. First, we computed permutation importance using the ranger package (500 trees, 1000 permutations). Second, we conducted stability selection with 200 bootstrap resamples (with replacement, sample size equal to the original dataset). These analyses provide complementary evidence on the reliability of the variable importance rankings derived from the original Gini−based method.

#### 2.4.3 Definition of core FAs intersection subsets

Three FA subsets of interest were then defined based on the intersection between statistical significance and model-derived importance:

1. Infertility_Sig = Set_Sig ∩ Imp_RF1 (FAs that are both statistically significant and important for distinguishing infertility from NC).
2. RSA_Sig = Set_Sig ∩ Imp_RF2 (FAs that are both statistically significant and important for distinguishing RSA from NC).
3. Infertility + RSA_Sig = Set_Sig ∩ Imp_RF1 ∩ Imp_RF2 (FAs that are simultaneously significant in univariate analysis and important in both classification tasks).

This intersection strategy ensures that each retained subset reflects both robust statistical evidence and predictive relevance for the respective disease comparison.

#### 2.4.4 Association between FAs and aPL positivity

Binary logistic regression was used to evaluate associations between aPL positivity and core FAs within each outcome−specific subset (infertility subset: NC + infertility; RSA subset: NC + RSA). Crude models and models adjusted for age and BMI were fitted. All continuous variables were standardized to z−scores; thus, odds ratios correspond to a 1−standard deviation increment. Adjusted confidence intervals were obtained via nonparametric bootstrapping (1,000 resamples). Results are presented as a combined table and forest plot. Analyses were performed using the glm, boot, and forestplot packages.

#### 2.4.5 Mediation analysis

Mediation analysis was conducted to examine whether aPL positivity mediated the associations of the omega−6/omega−3 ratio and DPAn3 with reproductive outcomes. All models were adjusted for age and BMI. Given the three−category outcome, analyses were stratified into two binary contrasts: infertility vs. NC, and RSA vs. NC. For each contrast, a structural equation model was specified with two equations: (1) binary aPL regressed on the FAs, age, and BMI; and (2) the binary outcome regressed on the FAs, aPL, age, and BMI. Continuous predictors were standardized. Indirect effects (*a* × *b*), direct effects (*c’*), and total effects (*c* = *c’* + *a* × *b*) were estimated, and the proportion mediated was calculated as the ratio of indirect to total effect. P−values for all parameters were derived from the WLSMV−fitted model via the delta method, and statistical significance of indirect effects was determined at P < 0.05. Nonparametric bootstrapping with 1,000 resamples was used to derive 95% confidence intervals for all effects to provide interval estimates. Analyses were performed using the lavaan package (v0.6−19) with the WLSMV estimator for binary outcomes.

## 3 Results

### 3.1 Study population and baseline characteristics

After excluding participants with autoimmune or metabolic disorders, reproductive structural abnormalities, male factor infertility, known chromosomal anomalies, or recent use of interfering medications, a total of 388 women were included. Reproductive histories of the three groups are summarized as follows. Among the 64 normal controls, 52 were aged ≥ 30 years, all of whom had at least one live birth (50 with one live birth, 2 with two live births); the remaining 12 controls aged < 30 years had no live births or adverse pregnancy history. In the infertility group (n = 135), 91 women had primary infertility and 44 had secondary infertility. The duration of infertility was: < 2 years (18), 2–3 years (32), 3–4 years (26), 4–5 years (19), > 5 years (23), and not clearly recorded (17). In the RSA group (n = 189), 127 women had primary RSA and 62 had secondary RSA. The number of miscarriages was: 2 (84), 3 (48), 4 (32), ≥ 5 (19), and not clearly recorded (6).

Age and BMI were comparable among the three groups (*P* = 0.63 and *P* = 0.91, respectively). Significant differences were observed for several FAs (omega-3, EPA, DPAn3, DHA, adrenic acid, pentadecanoic acid, behenic acid, lignoceric acid, omega-6/omega-3 ratio, EPA/AA ratio, and SFA/UFA ratio; all *P* < 0.05). The rate of aPL positivity also differed significantly across groups (9.4% vs. 25.9% vs. 30.7%, *P* = 0.003). Baseline characteristics are summarized in **Table 1**.

**Table 1:** Baseline characteristics of the study participants.

Variable	NC (n = 64)	Infertility (n = 135)	RSA (n = 189)	P value
Demographics (mean ± SD)				
Age (years)	34.2 ± 4.8	34.1 ± 4.4	34.6 ± 4.6	0.63
BMI (kg/m²)	21.5 ± 1.1	21.4 ± 1.1	21.4 ± 1.1	0.91
FAs [%, median (IQR)]				
Omega-3	5.97 (5.05-6.82)	5.01 (4.43-5.66)	5.33 (4.59-6.02)	< 0.001
ALA	0.65 (0.46-0.85)	0.53 (0.42-0.72)	0.56 (0.41-0.75)	0.108
EPA	0.41 (0.33-0.54)	0.30 (0.23-0.43)	0.34 (0.25-0.46)	< 0.001
DPAn3	1.48 (1.27-1.72)	1.29 (1.12-1.48)	1.27 (1.12-1.55)	< 0.001
DHA	3.24 (2.52-3.99)	2.81 (2.34-3.31)	2.91 (2.47-3.40)	0.004
Omega-6	35.09 (33.53-36.71)	34.97 (33.34-36.88)	35.23 (34.20-37.17)	0.196
LA	19.93 (18.91-21.43)	20.11 (18.78-21.82)	20.40 (19.11-21.78)	0.381
GLA	0.15 (0.11-0.26)	0.13 (0.09-0.21)	0.16 (0.09-0.23)	0.252
DGLA	1.49 (1.21-1.74)	1.47 (1.28-1.65)	1.47 (1.28-1.67)	0.934
AA	11.09 (9.64-12.30)	10.47 (9.59-11.61)	10.95 (9.85-12.17)	0.127
Adrenic acid	1.80 (1.54-2.05)	1.72 (1.47-2.00)	1.74 (1.54-1.99)	0.473
DPAn6	0.57 (0.43-0.65)	0.49 (0.40-0.57)	0.49 (0.40-0.59)	0.029
cis-MUFAs	19.03 (17.20-20.74)	18.89 (17.31-20.52)	18.59 (17.10-20.02)	0.304
Tetradecenoic acid	0.00 (0.00-0.00)	0.00 (0.00-0.00)	0.00 (0.00-0.00)	0.678
Pentadecenoic acid	0.02 (0.00-0.09)	0.03 (0.00-0.11)	0.04 (0.00-0.10)	0.555
Palmitoleic acid	0.58 (0.39-0.75)	0.56 (0.42-0.74)	0.54 (0.41-0.69)	0.314
Heptadecenoic acid	0.45 (0.39-0.53)	0.44 (0.37-0.52)	0.44 (0.35-0.55)	0.903
Oleic acid	15.07 (13.53-16.23)	14.90 (13.86-16.34)	14.64 (13.65-15.83)	0.171
Eicosenoic acid	0.30 (0.25-0.36)	0.29 (0.24-0.34)	0.28 (0.23-0.35)	0.547
Erucic acid	0.32 (0.22-0.41)	0.26 (0.19-0.43)	0.25 (0.19-0.39)	0.172
Nervonic acid	2.11 (1.40-2.52)	1.86 (1.51-2.37)	2.03 (1.61-2.48)	0.258
SFAs	37.66 (35.95-38.96)	38.52 (37.30-39.94)	38.26 (36.99-39.39)	0.006
Myristic acid	0.23 (0.16-0.31)	0.21 (0.15-0.30)	0.19 (0.13-0.28)	0.046
Pentadecanoic acid	0.19 (0.13-0.25)	0.15 (0.10-0.21)	0.15 (0.10-0.20)	0.002
Palmitic acid	20.40 (18.93-21.68)	20.66 (19.53-21.89)	20.24 (19.18-21.51)	0.129
Margaric acid	0.33 (0.29-0.36)	0.33 (0.29-0.37)	0.34 (0.30-0.38)	0.205
Stearic acid	13.16 (11.98-14.35)	13.85 (12.72-14.68)	13.61 (12.54-14.83)	0.168
Arachidic acid	0.31 (0.26-0.36)	0.32 (0.28-0.36)	0.32 (0.29-0.37)	0.065
Behenic acid	0.95 (0.75-1.15)	1.00 (0.84-1.18)	1.04 (0.90-1.25)	0.023
Lignoceric acid	1.56 (1.20-2.07)	1.67 (1.34-1.99)	1.75 (1.42-2.20)	0.021
Omega-6/Omega-3	6.19 (5.18-6.86)	7.01 (6.21-7.82)	6.80 (5.82-7.64)	< 0.001
EPA/AA	0.04 (0.03-0.05)	0.03 (0.02-0.04)	0.03 (0.02-0.04)	< 0.001
SFA/UFA	0.63 (0.58-0.66)	0.65 (0.62-0.69)	0.64 (0.61-0.67)	0.009
Immunology				
aPL positive, n (%)	6 (9.4)	35 (25.9)	58 (30.7)	0.003

Data are presented as mean ± standard deviation (SD), median (interquartile range, IQR), or number (%). P values were calculated using one−way ANOVA (age, BMI), Kruskal−Wallis test (FAs), or Fisher’s exact test (aPL). Bold values indicate statistical significance (P < 0.05). NC, normal control; FAs, fatty acids; RSA, recurrent spontaneous abortion; aPL, antiphospholipid antibody.

### 3.2 Overall and univariate analyses of FAs

After adjusting for age and BMI, MANCOVA revealed a significant overall difference in FAs composition among the three groups (Pillai’s trace = 0.228, F = 1.374, *P* = 0.030). Subsequent univariate analyses (ANCOVA for normally distributed FAs; Kruskal–Wallis test on age− and BMI−adjusted residuals for non−normally distributed FAs) identified 9 FAs with significant group differences after FDR correction (q < 0.10): omega−3, omega−6/omega−3 ratio, DHA, DPAn3, SFA/UFA ratio, EPA, GLA, behenic acid, and total SFAs. This set (Set_Sig) was retained for further refinement by random forest analysis. Full results are provided in **Table 2**.

**Table 2:** Univariate analysis of FAs among NC, infertility, and RSA groups.

FAs	P value	Q-value
Omega-3	< 0.001	< 0.001
Omega-6/Omega-3	< 0.001	< 0.001
DHA	0.001	0.011
DPAn3	0.006	0.050
SFA/UFA	0.012	0.079
EPA	0.018	0.099
GLA	0.021	0.099
Behenic acid	0.023	0.095
SFAs	0.025	0.092
Arachidic acid	0.033	0.109
Oleic acid	0.036	0.108
Adrenic acid	0.046	0.127
Palmitic acid	0.084	0.213
Palmitoleic acid	0.092	0.217
cis-MUFAs	0.095	0.209
Myristic acid	0.105	0.217
Omega-6	0.118	0.229
ALA	0.123	0.2255
EPA/AA	0.139	0.241
Stearic acid	0.207	0.342
AA	0.211	0.332
Lignoceric acid	0.321	0.482
Nervonic acid	0.341	0.489
Margaric acid	0.458	0.630
LA	0.477	0.630
Eicosenoic acid	0.525	0.666
Heptadecenoic acid	0.557	0.681
DPAn6	0.612	0.721
Pentadecenoic acid	0.624	0.710
Pentadecanoic acid	0.655	0.721
DGLA	0.705	0.750
Tetradecenoic acid	0.738	0.761
Erucic acid	0.868	0.868

P values were obtained from ANCOVA (normally distributed FAs) or Kruskal–Wallis test on age− and BMI−adjusted residuals (non−normally distributed FAs). q values represent false discovery rate (FDR) adjusted P values using the Benjamini–Hochberg method. FAs with q < 0.10 (in bold) were retained as the candidate set (Set_Sig) for subsequent random forest analysis. FAs, fatty acids.

### 3.3 Random forest importance

**Figure 1:**
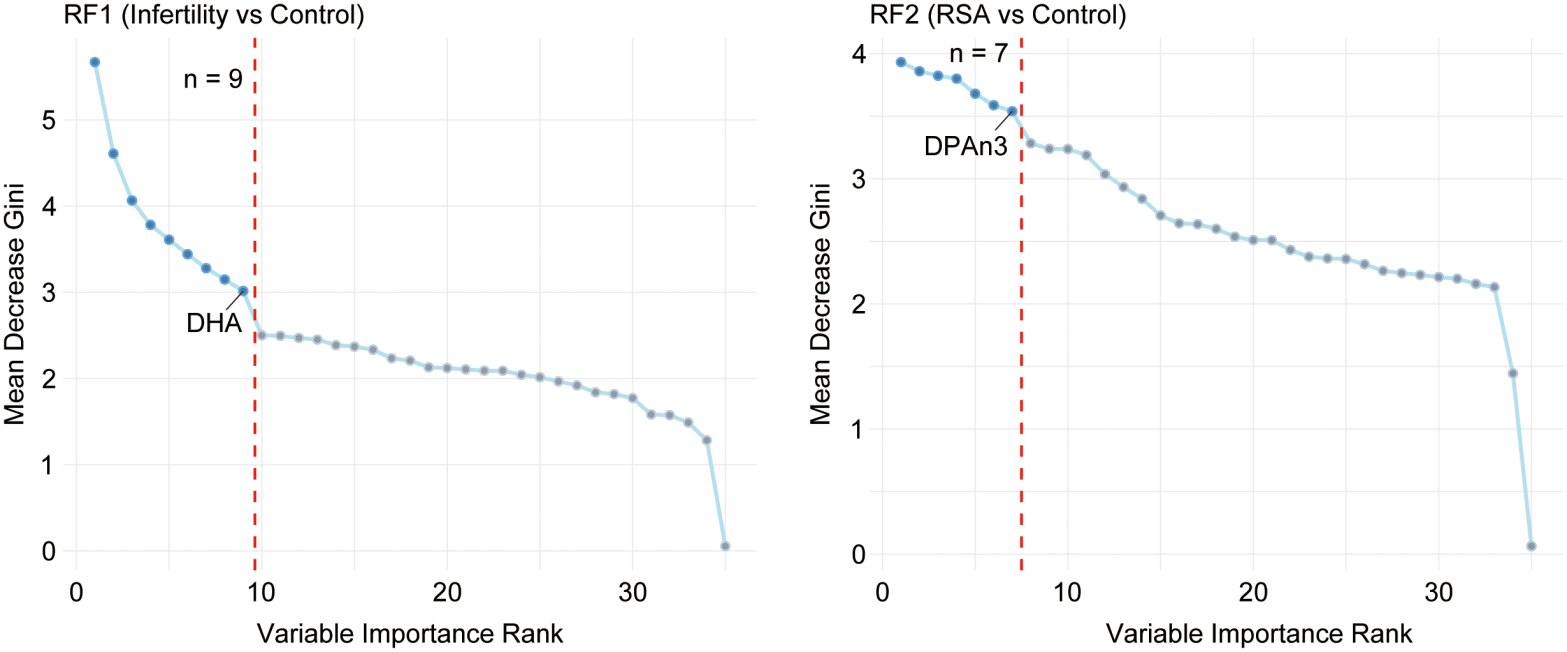
Elbow point determination for random forest models. Importance curves for RF1 (infertility vs. control) and RF2 (RSA vs. control) are shown. The elbow points (marked by dashed red lines) are located at the 9th variable (DHA) for RF1 and the 7th variable (DPAn3) for RF2, beyond which the importance declines more slowly. RSA, recurrent spontaneous abortion.

To further explore which of the statistically significant FAs contribute more to group discrimination, two random forest classification models (RF1 and RF2) were constructed. The variable importance ranking curves are presented in **Figure 1**. In the RF1 model, the mean importance across all predictors was 2.457, and the importance curve showed a clear elbow point at the 9th variable; the top 9 variables above this elbow point were retained as the important variable set Imp_RF1. In the RF2 model, the mean importance across all predictors was 2.712, and the elbow point was located at the 7th variable; the top 7 variables above this elbow point were retained as Imp_RF2. The variable importance rankings for both models are presented in **Table 3**, together with the AUC values (0.727 for RF1 and 0.594 for RF2.

**Table 3:** Variable importance rankings from random forest models for infertility and RSA.

RF1 (infertility)		RF2 (RSA)	
FAs	Importance (mean = 2.457)	FAs	Importance (mean = 2.712)
Omega-3	5.67090277	DPAn6	3.93198027
DPAn6	4.60891835	Arachidic acid	3.85929576
Omega-6/Omega-3	4.06591051	Pentadecanoic acid	3.82331395
EPA	3.78274918	Behenic acid	3.80007907
EPA/AA	3.61048875	Omega-6/Omega-3	3.67949704
Pentadecanoic acid	3.44258986	EPA/AA	3.58767202
DPAn3	3.27974902	DPAn3	3.53926048
Stearic acid	3.14910897	Lignoceric acid	3.28323027
DHA	3.01513325	Omega-3	3.23871546
DGLA	2.50121515	DHA	3.23755596
Nervonic acid	2.49564723	GLA	3.18872576
Behenic acid	2.47088926	Heptadecenoic acid	3.03607929
SFAs/UFAs	2.45228771	EPA	2.93359212
Lignoceric acid	2.38755818	SFAs	2.83937053
SFAs	2.36996531	Myristic acid	2.70675252
Arachidic acid	2.33400853	Oleic acid	2.64379954
Palmitic acid	2.23437005	Stearic acid	2.63665412
Erucic acid	2.20746458	SFAs/UFAs	2.59955956
Myristic acid	2.12974487	Palmitic acid	2.53666052
ALA	2.10629331	DGLA	2.50983263
cis-MUFAs	2.09070577	Adrenic acid	2.50830815
AA	2.09037884	Palmitoleic acid	2.43084185
LA	2.01492345	LA	2.37684175
Eicosenoic acid	1.96668604	ALA	2.36296242
Oleic acid	1.92013651	Erucic acid	2.35862795
Heptadecenoic acid	1.83984981	cis-MUFAs	2.31728764
Adrenic acid	1.81890667	Eicosenoic acid	2.26394921
GLA	1.77266095	Nervonic acid	2.24577511
Palmitoleic acid	1.58279618	AA	2.23160365
Margaric acid	1.57451036	Margaric acid	2.213703
Omega-6	1.48975094	Omega-6	2.20091235
Pentadecenoic acid	1.28370969	Pentadecenoic acid	2.15970468
Tetradecenoic acid	0.05502813	Tetradecenoic acid	2.13394973

Variables are ranked in descending order of Mean Decrease Gini. For RF1 (infertility vs. normal controls), the mean importance across all predictors was 2.457, and the top 9 variables above the elbow point at DHA were retained as Imp_RF1, with an AUC of 0.727. For RF2 (RSA vs. NC), the mean importance was 2.712, and the top 7 variables above the elbow point at DPAn3 were retained as Imp_RF2, with an AUC of 0.594. FAs, fatty acids; RSA, recurrent spontaneous abortion.

Sensitivity analyses (SMOTE-balanced Gini, permutation importance, and 200-bootstrap stability) confirmed that core FAs (omega-6/omega-3 ratio, DPAn3, EPA) remained important in both RF1 and RF2. Behenic acid showed reduced importance in RSA, supporting cautious interpretation. Detailed results are provided in **Supplementary Tables 1**–**3**.

### 3.4 Core FAs for infertility and RSA

**Figure 2:**
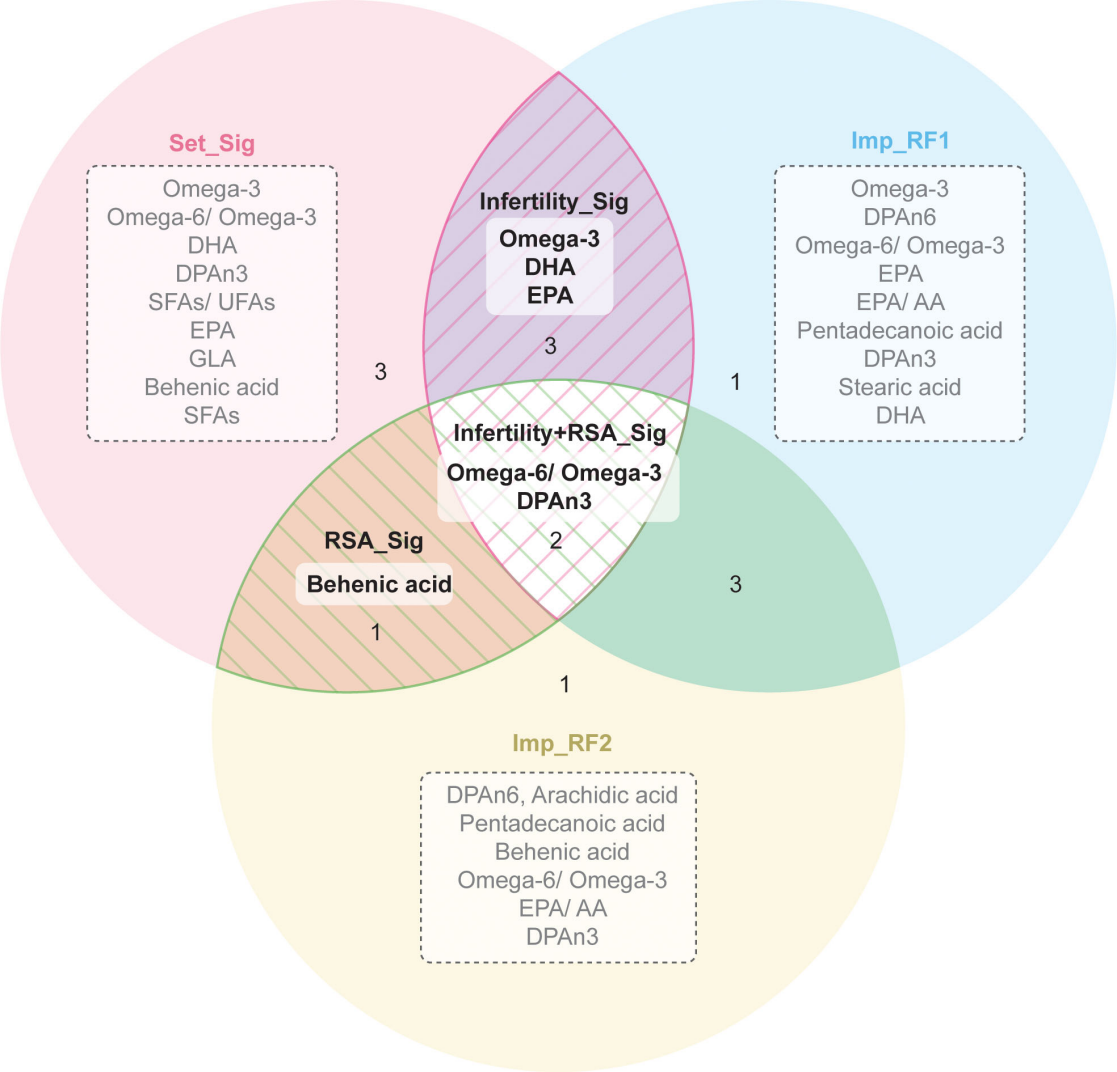
Overlap of candidate FAs with random forest important variables. Venn diagram showing the intersection of FDR-significant FAs (Set_Sig) with important variables from random forest models for infertility (Imp_RF1) and RSA (Imp_RF2). Overlapping regions indicate FAs relevant to each outcome contrast. FAs, fatty acids; RSA, recurrent spontaneous abortion.

To identify FAs associated with each reproductive outcome, the candidate set from FDR screening (Set_Sig) was intersected with important variables from the random forest models for infertility (RF1) and RSA (RF2). As shown in **Figure 2**, this process defined the core FAs sets for subsequent analyses: the infertility core set comprised omega-3, DHA, EPA, DPAn3, and the omega-6/omega-3 ratio; the RSA core set comprised behenic acid, DPAn3, and the omega-6/omega-3 ratio. The omega-6/omega-3 ratio and DPAn3 were common to both sets.

### 3.5 Association of core FAs with aPL status

**Figure 3:**
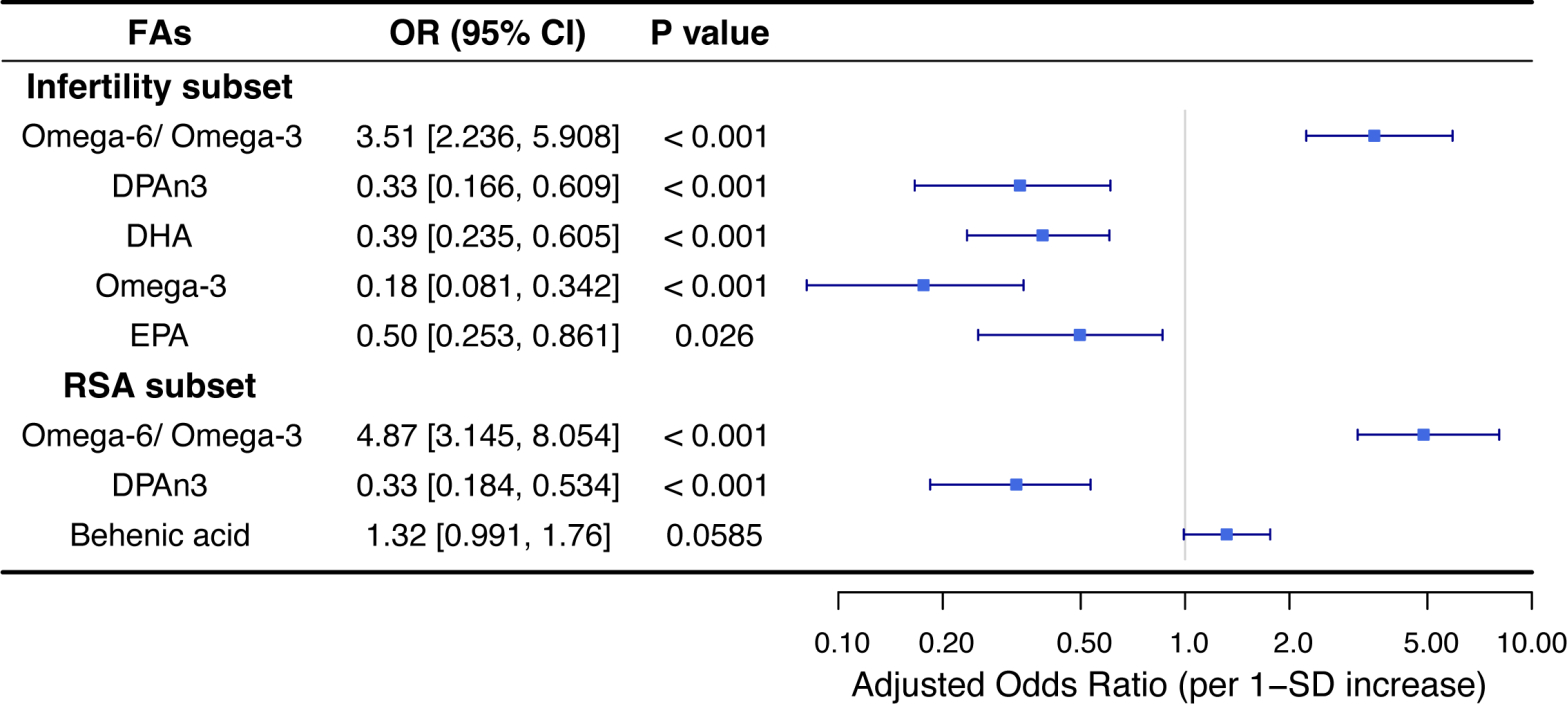
Associations between core FAs and aPL positivity. Forest plot displaying adjusted ORs and 95% CIs for aPL positivity per 1-SD deviation increase in each FA. Models were adjusted for age and BMI. The vertical dashed line denotes an OR of 1. FAs, fatty acids; RSA, recurrent spontaneous abortion.

We examined the associations between core FAs and aPL positivity within each outcome-specific subset after adjusting for age and BMI (**Figure 3**). In the infertility subset, the omega-6/omega-3 ratio was positively associated with aPL positivity (adjusted OR = 3.51, 95% CI: 2.24–5.91, *P* < 0.001), whereas omega-3, EPA, DPAn3, and DHA showed significant inverse associations (all *P* < 0.05). In the RSA subset, the omega-6/omega-3 ratio was also strongly and positively associated with aPL positivity (adjusted OR = 4.87, 95% CI: 3.15–8.05, *P* < 0.001), while DPAn3 exhibited a significant inverse association (adjusted OR = 0.33, 95% CI: 0.18–0.53, *P* < 0.001). Behenic acid showed a positive trend that did not reach statistical significance (*P* = 0.059). Given its consistent and robust positive associations across both subsets, the omega-6/omega-3 ratio was selected for subsequent mediation analysis.

### 3.6 Mediation analyses

Mediation analysis was performed to evaluate the potential mediating role of aPL positivity in the associations of the omega-6/omega-3 and DPAn3 with reproductive outcomes, adjusting for age and BMI. For the omega-6/omega-3 ratio, the indirect effect on infertility was not significant (*P* = 0.361), whereas the indirect effect on RSA reached statistical significance (*P* = 0.045), with a proportion mediated of 30.7%. For DPAn3, the indirect effect on infertility was not significant (*P* = 0.069). In the RSA contrast, the indirect effect of DPAn3 was significant (*P* = 0.022), and the proportion mediated was 40.1%. Complete effect estimates, 95% confidence intervals, and *P* values are provided in **Table 4**. In summary, aPL positivity partially mediated the associations of both the omega-6/omega-3 ratio and DPAn3 with RSA, but did not significantly mediate their associations with infertility.

**Table 4:** Mediation effects of aPL positivity on the associations of omega-6/omega-3 ratio and DPAn3 with reproductive outcomes.

FAs	Outcome	Effect	Estimate	95% CI	P
Omega 6/Omega 3	Infertility vs NC	Indirect effect	0.042	-0.043, 0.158	0.361
		Direct effect	0.500	0.28, 0.786	<0.001
		Total effect	0.542	0.366, 0.828	<0.001
		Proportion	0.078	-0.082, 0.297	0.368
	RSA vs NC	Indirect effect	0.110	0.007, 0.237	0.0453
		Direct effect	0.249	0.051, 0.462	0.0325
		Total effect	0.359	0.196, 0.543	<0.001
		Proportion	0.307	0.024, 0.783	0.0795
DPAn3	Infertility vs NC	Indirect effect	-0.053	-0.13, -0.007	0.0691
		Direct effect	-0.226	-0.653, -0.035	<0.001
		Total effect	-0.279	-0.728, -0.088	<0.001
		Proportion	0.189	0.014, 0.648	0.0427
	RSA vs NC	Indirect effect	-0.080	-0.154, -0.03	0.0218
		Direct effect	-0.119	-0.314, 0.055	0.1716
		Total effect	-0.198	-0.384, -0.031	0.0207
		Proportion	0.401	0.11, 2.068	0.0741

Models were adjusted for age and BMI. All continuous variables were standardized prior to analysis; estimates represent standardized regression coefficients. P−values were derived from the WLSMV−fitted model via the delta method, with statistical significance set at P < 0.05. 95% confidence intervals were obtained via nonparametric bootstrapping (1,000 resamples) and are presented as interval estimates. FAs, fatty acids; RSA, recurrent spontaneous abortion; NC, normal control.

## 4 Discussion

**Figure 4:**
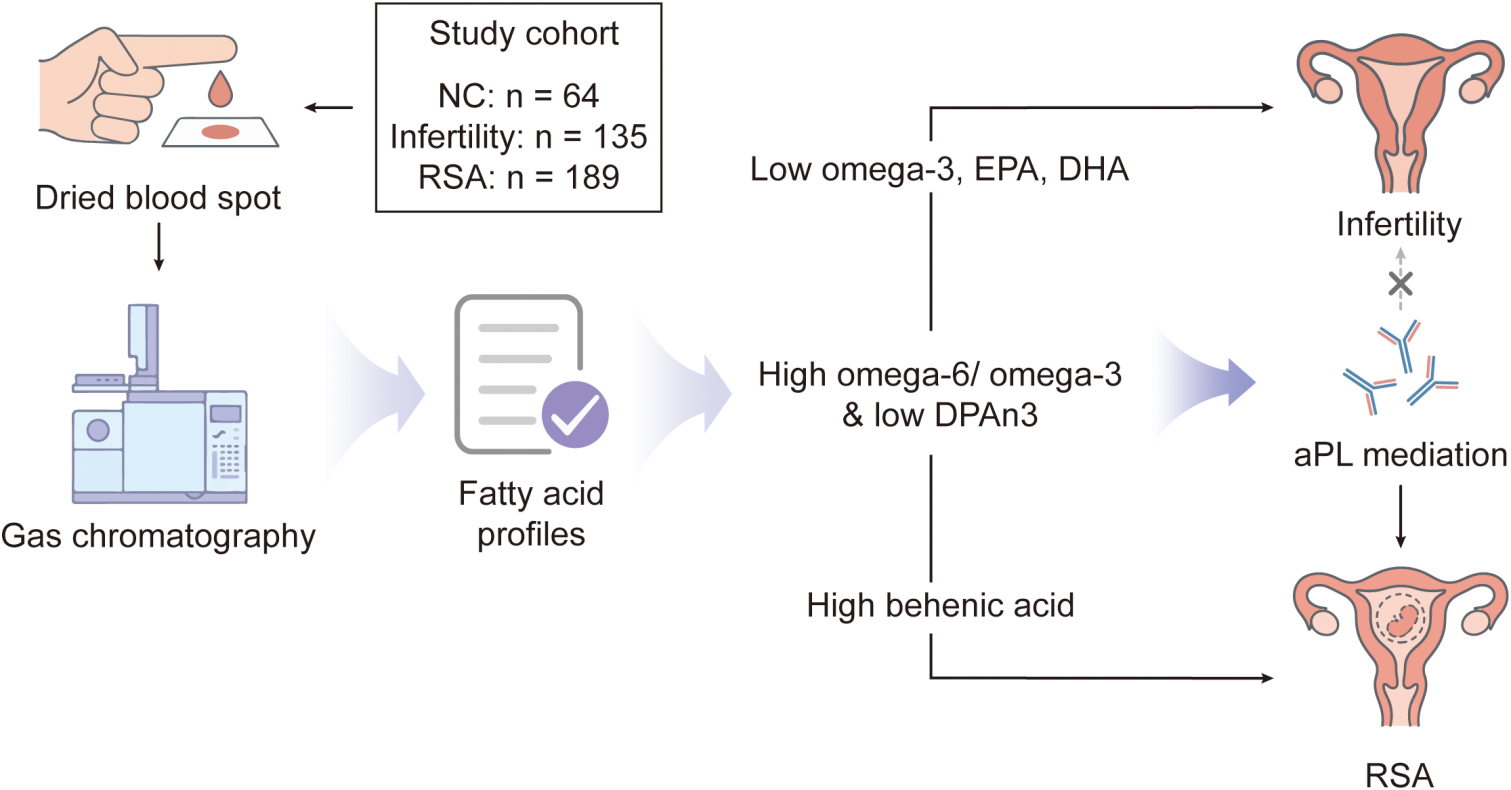
Comparison of FA profiles among normal controls (n = 64), infertility (n = 135), and RSA (n = 189). Dried blood spot samples were analyzed by gas chromatography. Both disease groups shared an elevated omega-6/omega-3 ratio and low DPAn3; infertility additionally displayed low omega-3, EPA, and DHA, while RSA exhibited high behenic acid. The associations of omega-6/omega-3 ratio and DPAn3 with RSA were partially mediated by aPL positivity. aPL, antiphospholipid antibody; NC, normal controls; RSA, recurrent spontaneous abortion.

This retrospective study examined FA profiles measured by DBS from fingertip blood samples in three groups: women with infertility, women with RSA, and normal controls. Multivariate screening, RF ranking, and mediation analysis were used to evaluate the mediating role of aPL positivity. Both disease groups exhibited distinct shifts in FA composition. An elevated omega−6/omega−3 ratio and reduced DPAn3 were common to both infertility and RSA. aPL positivity partially mediated the associations of these FA indices with RSA, but no such mediation was observed for infertility (**Figure 4**).

The role of FA metabolism in reproductive health has received increasing attention. FAs serve as essential structural components of cell membrane phospholipids, and their derivatives participate in regulating inflammation, oxidative stress, and immune cell function (5). They are therefore critical for follicular development, embryo implantation, and pregnancy maintenance. DBS sampling from fingertip blood offers practical advantages, including minimal sample volume, ease of collection, and FA stability during ambient storage and transport. This technique has been applied in nutritional epidemiology and population−based studies for FA profiling (18) (19), although its use in reproductive research remains limited. In this study, women with infertility and RSA showed overlapping yet distinct FA imbalance profiles. An elevated omega−6/omega−3 ratio and reduced DPAn3 were common to both groups. The infertility group exhibited a broader decline in omega−3−related FAs, whereas the RSA group was characterized by increased behenic acid.

In infertile women, core FA abnormalities mainly involved omega−3−related components. Lower levels of omega−3, EPA, DHA, and DPAn3 were accompanied by an elevated omega−6/omega−3 ratio. This pattern suggests a relative deficiency of anti−inflammatory omega−3 FAs and an imbalance in PUFAs metabolism. Omega−3 regulates multiple aspects of female reproduction. In women with diminished ovarian reserve, supplementation with flaxseed oil—a rich source of α-linolenic acid—significantly increased the MII oocyte rate, fertilization rate, cleavage rate, high-quality embryo rate, and blastocyst formation rate; moreover, flaxseed oil intake was an independent predictor of higher MII oocyte rate (20) (21). EPA and DHA are critical membrane constituents that may influence sperm-oocyte fusion, calcium oscillations, and embryonic cleavage (22). In a PCOS rat model, high-dose omega-3 supplementation combined with metformin improved endometrial receptivity markers, including increased integrin β3 expression, decreased MUC-1 expression, and enhanced pinopode-like structure formation (23). Clinical evidence also suggests that omega-3 FAs may serve as adjunctive agents to improve endometrial receptivity in women with PCOS (24). DPAn3 serves as a key intermediate in the conversion of EPA to DHA; its reduction could limit DHA bioavailability (25). Collectively, these FA alterations may compromise fertility by disrupting follicular development, impairing fertilization and embryonic competence, and reducing endometrial receptivity. None of these core FAs showed significant indirect effects via aPL positivity, suggesting that their impact on infertility is independent of aPL−related immune pathways.

In the RSA group, core FA abnormalities included reduced DPAn3, an elevated omega−6/omega−3 ratio, and increased behenic acid. The overlap of DPAn3 and the omega−6/omega−3 ratio across groups points to a shared metabolic disturbance. Behenic acid emerged as a core feature only in RSA, indicating potential relevance to pregnancy maintenance. Omega−3, particularly DHA and EPA, contribute to early placentation by protecting against disrupted angiogenesis and promoting trophoblast invasion via MMP9 expression (26). DHA insufficiency, by contrast, impairs placental angiogenesis through inhibition of AKT−VEGFA signaling (27). Optimal maternal omega−3 intake also supports uteroplacental vascular remodeling and maintains placental structural integrity (28), whereas n−3 deficiency alters uterine artery morphology and disrupts vascular marker expression (29). In a mouse model, a maternal diet low in omega-3—and thus high in omega-6/omega-3 ratio—led to hyperlipidemia and reduced fetal numbers (30). Behenic acid, a very−long−chain SFA, has been linked to several pregnancy−related disorders. Metabolomic profiling identified behenic acid as a characteristic metabolite distinguishing gestational diabetes mellitus and preeclampsia from normal pregnancy (31). Higher circulating behenic acid levels were associated with a reduced risk of pregnancy−induced hypertension (32). In a mouse model of gestational diabetes mellitus (GDM), behenic acid alleviated inflammation and insulin resistance by inhibiting the TLR4/NF−κB pathway (33). A recent meta-analysis of 25 studies demonstrated that circulating behenic acid levels were significantly lower in women with gestational diabetes mellitus compared with normal pregnant women, and higher behenic acid concentrations were associated with a reduced risk of GDM (34). Thus, higher behenic acid levels correlate with lower risks of GDM, preeclampsia, and other pregnancy−related disorders, possibly via anti−inflammatory mechanisms. However, we found elevated behenic acid in RSA patients in this study. This difference likely reflects distinct disease mechanisms. The behenic acid accounts for only ~1% of circulating FAs, so its biological effect may be limited and the elevation might simply be an epiphenomenon of overall metabolic dysregulation. We speculate that elevated behenic acid in RSA represents a compensatory protective response (upregulating to suppress inflammation, but insufficient to prevent loss). This hypothesis is consistent with its anti−inflammatory effects and requires further functional validation.

It should be noted that the control group was smaller than the disease groups, potentially introducing class imbalance. However, three sensitivity analyses—SMOTE−balanced Gini importance, permutation importance, and 200−bootstrap stability—all confirmed that the core FAs (omega−6/omega−3 ratio, DPAn3) remained important for RSA, while behenic acid consistently showed reduced importance. Thus, our main conclusion is unlikely to be driven by class imbalance, but the finding on behenic acid should be interpreted cautiously and validated in larger cohorts.

Both DPAn3 and the omega−6/omega−3 ratio in the RSA group were significantly associated with aPL positivity. Mediation analysis indicated that aPL positivity partially mediated their associations with RSA. Notably, aPL positivity in this study was defined by a single positive test result. According to established criteria, a diagnosis of APS requires persistent aPL positivity on at least two occasions 12 weeks apart (15). Therefore, the present findings do not establish a direct link between FA dysregulation and clinically defined APS. Instead, they raise the possibility that FA imbalance may create a metabolic environment that favors aPL production or enhances its pathogenic effects, thereby increasing vulnerability to pregnancy loss. In this regard, it is noteworthy that in a series of 22 women with persistent APS and RSA, supplementation with fish oil providing 5.1 g of EPA and DHA daily resulted in 22 live births out of 23 pregnancies over three years, with no adverse reactions reported (35). Although encouraging, this observation derives from an uncontrolled case series and should be interpreted with caution. Whether similar associations occur in women with confirmed APS requires further study in larger, prospective cohorts.

In this study, several limitations should be noted. First, the retrospective, single−center design is a fundamental limitation that entirely precludes causal inference and limits generalizability. Future cohort studies are needed to further explore changes before and after specific FA supplementation. Second, DBS−based FA measurements reflect a single time point and cannot capture intra−individual variability or long−term dietary patterns. Although all participants maintained their usual diet and avoided FA supplements for ≥ 3 months before sampling, dietary intake, supplement use, and other lifestyle factors were not directly measured, which remains a central limitation of our study. Future studies using dietary frequency questionnaires or objective physical activity monitors could help address the confounding effects of unmeasured lifestyle factors. Finally, aPL positivity was defined by a single test rather than confirmed persistent positivity, which may have included transient or false−positive cases and could attenuate or inflate the observed mediation effects. Finally, the indirect effect of the omega−6/omega−3 ratio on RSA via aPL positivity reached only borderline statistical significance and should be interpreted cautiously pending confirmation in larger independent cohorts.

In summary, infertility and RSA shared a pattern of higher omega-6/omega-3 ratio and lower DPAn3. Beyond this common signature, omega-3, DHA, and EPA were more relevant to infertility, and behenic acid to RSA. In RSA, aPL positivity partially mediated the relationship between the omega−6/omega−3 ratio and DPAn3 with RSA, suggesting that dysregulation of these FAs is associated with RSA in part through aPL−related pathways.

## Data Availability

The data that support the findings of this study are provided in **Supplementary Table 4**. Further information is available from the corresponding author upon reasonable request.

## Supplementary material

Supplementary Table 1 Variable importance rankings from SMOTE−balanced random forest models for infertility and RSA.

Supplementary Table 2 Permutation importance of fatty acids for RF1 and RF2 models (original).

Supplementary Table 3 Stability of variable selection assessed by 200 bootstrap resamples.
