# Supplementary material for: Overlapping and distinct fatty acid dysregulation in infertility and recurrent spontaneous abortion
Source: Front Endocrinol (Lausanne). 2026 Jun 12;17:1866902. doi: 10.3389/fendo.2026.1866902 (PMC13303191; doi:10.3389/fendo.2026.1866902)
Supplement: Supplementary Table 1 — Variable importance rankings from SMOTE−balanced random forest models for infertility and RSA. [file Table1.docx]

**Table S1.** Variable importance rankings from SMOTE‑balanced random forest models for infertility and RSA.

| **RF1 (infertility)** | | **RF2 (RSA)** | |
| --- | --- | --- | --- |
| FAs | Importance  （mean = 4.343） | FAs | Importance  （mean = 3.741） |
| EPA/ AA | 8.643490753 | Omega-3 | 9.584516091 |
| Pentadecanoic acid | 8.495301353 | EPA | 9.412308365 |
| EPA | 6.616420238 | Omega-6/ Omega-3 | 7.320546106 |
| Omega-6/ Omega-3 | 6.533760358 | EPA/ AA | 7.220256884 |
| DPAn6 | 6.238693406 | DPAn6 | 6.733996133 |
| DPAn3 | 6.18870017 | Pentadecanoic acid | 5.855658114 |
| GLA | 5.714899519 | Erucic acid | 5.06851314 |
| Erucic acid | 5.693493465 | DHA | 4.343430267 |
| Myristic acid | 5.687873526 | DPAn3 | 4.071989122 |
| Omega-3 | 5.575714128 | SFA/ UFA | 3.97643479 |
| Arachidic acid | 5.167845383 | DGLA | 3.729244717 |
| Heptadecenoic acid | 4.246417266 | SFAs | 3.723692835 |
| Eicosenoic acid | 4.232094053 | Stearic acid | 3.66301786 |
| DHA | 4.126463395 | ALA | 3.646313408 |
| Lignoceric acid | 4.077653674 | Myristic acid | 3.316815119 |
| ALA | 4.014361474 | Nervonic acid | 3.098138702 |
| SFAs | 3.949180891 | cis-MUFAs | 3.042625442 |
| Palmitic acid | 3.875346574 | Arachidic acid | 2.892603254 |
| Behenic acid | 3.78483024 | Oleic acid | 2.87329578 |
| DGLA | 3.730644424 | GLA | 2.836542357 |
| Stearic acid | 3.653431337 | Eicosenoic acid | 2.795128012 |
| SFA/ UFA | 3.623442549 | Margaric acid | 2.753814602 |
| LA | 3.556440056 | Adrenic acid | 2.719718872 |
| Oleic acid | 3.518495905 | LA | 2.625701809 |
| Margaric acid | 3.48909214 | Palmitic acid | 2.609049457 |
| cis-MUFAs | 3.471311644 | Palmitoleic acid | 2.608368547 |
| Palmitoleic acid | 3.422450392 | Omega-6 | 2.592345087 |
| Omega-6 | 3.375816625 | Lignoceric acid | 2.552381281 |
| Adrenic acid | 3.221227845 | AA | 2.49032139 |
| Nervonic acid | 3.195010807 | Heptadecenoic acid | 2.417635316 |
| AA | 3.143292811 | Behenic acid | 2.326216104 |
| Pentadecenoic acid | 2.992254801 | Pentadecenoic acid | 2.177993175 |
| Tetradecenoic acid | 2.865637256 | Tetradecenoic acid | 2.113690922 |

Variables ranked by Mean Decrease Gini (descending). To address class imbalance, SMOTE (K = 5) was applied before model training. Cross‑validated AUCs: 0.723 (RF1_SMOTE) and 0.63 (RF2_SMOTE). Rankings for infertility were consistent with the original model. For RSA, omega‑6/ omega‑3 ratio and DPAn3 remained in the top variables, whereas behenic acid decreased in importance. FAs: fatty acids; RSA: recurrent spontaneous abortion; SMOTE: Synthetic Minority Over‑sampling Technique.
