# Supplementary material for: Overlapping and distinct fatty acid dysregulation in infertility and recurrent spontaneous abortion
Source: Front Endocrinol (Lausanne). 2026 Jun 12;17:1866902. doi: 10.3389/fendo.2026.1866902 (PMC13303191; doi:10.3389/fendo.2026.1866902)
Supplement: Supplementary Table 2 — Permutation importance of fatty acids for RF1 and RF2 models (original). [file Table2.docx]

**Table S2.** Permutation importance of fatty acids for RF1 and RF2 models (original).

| **RF1 (infertility)** | | **RF2 (RSA)** | |
| --- | --- | --- | --- |
| FAs | Permutation  importance | FAs | Permutation  importance |
| EPA | 0.0260 | Omega-6/Omega-3 | 0.0059 |
| Omega-3 | 0.0240 | DPAn6 | 0.0053 |
| EPA/AA | 0.0198 | EPA | 0.0042 |
| DPAn6 | 0.0198 | SFAs/UFAs | 0.0041 |
| Omega-6/Omega-3 | 0.0178 | Omega-3 | 0.0035 |
| Pentadecanoic acid | 0.0136 | SFAs | 0.0034 |
| DPAn3 | 0.0107 | EPA/AA | 0.0033 |
| Erucic acid | 0.0107 | Pentadecanoic acid | 0.0032 |
| SFAs | 0.0083 | Behenic acid | 0.0026 |
| Stearic acid | 0.0081 | Arachidic acid | 0.0023 |
| DHA | 0.0079 | Myristic acid | 0.0019 |
| ALA | 0.0076 | Omega-6 | 0.0018 |
| Myristic acid | 0.0067 | GLA | 0.0015 |
| DGLA | 0.0066 | DHA | 0.0013 |
| SFAs/UFAs | 0.0065 | cis-MUFAs | 0.0012 |
| cis-MUFAs | 0.0058 | Nervonic acid | 0.0011 |
| Palmitic acid | 0.0055 | DPAn3 | 0.0011 |
| GLA | 0.0046 | Palmitic acid | 0.0011 |
| Margaric acid | 0.0041 | Stearic acid | 0.0005 |
| Nervonic acid | 0.0040 | ALA | 0.0005 |
| Arachidic acid | 0.0040 | Margaric acid | 0.0005 |
| Adrenic acid | 0.0038 | Erucic acid | 0.0003 |
| Oleic acid | 0.0036 | Oleic acid | 0.0003 |
| AA | 0.0036 | Palmitoleic acid | 0.0003 |
| Omega-6 | 0.0035 | Heptadecenoic acid | 0.0002 |
| Eicosenoic acid | 0.0031 | DGLA | 0.0002 |
| LA | 0.0027 | LA | 0.0001 |
| Pentadecenoic acid | 0.0027 | Adrenic acid | 0.0001 |
| Heptadecenoic acid | 0.0025 | Tetradecenoic acid | 0.0000 |
| Palmitoleic acid | 0.0020 | AA | -0.0001 |
| Behenic acid | 0.0019 | Eicosenoic acid | -0.0001 |
| Lignoceric acid | 0.0019 | Lignoceric acid | -0.0001 |
| Tetradecenoic acid | 0.0018 | Pentadecenoic acid | -0.0005 |

Permutation importance was calculated using the ranger package with 500 trees and 1000 permutations. Values represent the mean decrease in prediction accuracy when each variable is randomly permuted. Variables are sorted in descending order of importance. FAs: fatty acids; RSA: recurrent spontaneous abortion.
