# Supplementary material for: Overlapping and distinct fatty acid dysregulation in infertility and recurrent spontaneous abortion
Source: Front Endocrinol (Lausanne). 2026 Jun 12;17:1866902. doi: 10.3389/fendo.2026.1866902 (PMC13303191; doi:10.3389/fendo.2026.1866902)
Supplement: Supplementary Table 3 — Stability of variable selection assessed by 200 bootstrap resamples. [file Table3.docx]

**Table S3.** Stability of variable selection assessed by 200 bootstrap resamples.

| **RF1 (infertility)** | | **RF2 (RSA)** | |
| --- | --- | --- | --- |
| FAs | Frequency | FAs | Frequency |
| Omega-3 | 98 | Behenic acid | 68.5 |
| Omega-6/Omega-3 | 93.5 | Pentadecanoic acid | 54 |
| DPAn6 | 89.5 | Arachidic acid | 54 |
| EPA | 80.5 | DPAn6 | 52.5 |
| EPA/AA | 73 | Omega-6/Omega-3 | 49.5 |
| DPAn3 | 65.5 | EPA/AA | 48.5 |
| Pentadecanoic acid | 55 | Omega-3 | 47 |
| Stearic acid | 52.5 | DHA | 44 |
| DHA | 46 | DPAn3 | 39.5 |
| SFAs | 40.5 | Lignoceric acid | 35 |
| SFAs/UFAs | 31 | Heptadecenoic acid | 26 |
| Behenic acid | 21 | GLA | 22.5 |
| DGLA | 18.5 | EPA | 21.5 |
| Nervonic acid | 18.5 | Myristic acid | 19 |
| Lignoceric acid | 18 | SFAs | 18 |
| Arachidic acid | 15.5 | Stearic acid | 14 |
| ALA | 13.5 | DGLA | 11.5 |
| AA | 13 | Palmitoleic acid | 11 |
| Erucic acid | 12 | SFAs/UFAs | 9.5 |
| Palmitic acid | 7 | Erucic acid | 9 |
| Eicosenoic acid | 6 | Nervonic acid | 7 |
| GLA | 5 | Eicosenoic acid | 6.5 |
| Myristic acid | 5 | Oleic acid | 6 |
| Oleic acid | 4 | LA | 4 |
| Adrenic acid | 4 | ALA | 4 |
| Palmitoleic acid | 3.5 | cis-MUFAs | 3.5 |
| Heptadecenoic acid | 2.5 | Adrenic acid | 3.5 |
| LA | 2 | Omega-6 | 3.5 |
| cis-MUFAs | 2 | AA | 3 |
| Omega-6 | 1.5 | Palmitic acid | 2 |
| Tetradecenoic acid | 1.5 | Tetradecenoic acid | 2 |
| Pentadecenoic acid | 1 | Pentadecenoic acid | 0.5 |
| Margaric acid | 0 | Margaric acid | 0 |

Based on 200 bootstrap resamples (with replacement, sample size equal to the original dataset), random forest models were trained for each bootstrap sample. The table shows the frequency (percentage) of each FA appearing among the top‑ranked variables across the 200 bootstrap iterations. Higher frequencies indicate more stable selection. FAs: fatty acids; RSA: recurrent spontaneous abortion.
